# Increasing Prevalence of Metabolic Syndrome in a Chinese Elderly Population: 2001–2010

**DOI:** 10.1371/journal.pone.0066233

**Published:** 2013-06-18

**Authors:** Miao Liu, Jianhua Wang, Bin Jiang, Dongling Sun, Lei Wu, Shanshan Yang, Yiyan Wang, Xiaoying Li, Yao He

**Affiliations:** 1 Institute of Geriatrics, Chinese PLA General Hospital, Beijing, China; 2 Beijing Key Laboratory of Aging and Geriatrics, Chinese PLA General Hospital, Beijing, China; 3 Department of Chinese Traditional Medicine and Acupuncture, Chinese PLA General Hospital, Beijing, China; 4 Department of Geriatric Cardiology, Chinese PLA General Hospital, Beijing, China; 5 State Key Laboratory of Kidney Disease, Chinese PLA General Hospital, Beijing, China; Innsbruck Medical University, Austria

## Abstract

**Objective:**

The information on the changes of prevalence of MetS in China is limited. Our objective was to assess a 10-year’s change of the prevalence of MetS in a Chinese elderly population between 2001 and 2010.

**Methods:**

We conducted two cross-sectional surveys in a representative sample of elderly population aged 60 to 95 years in Beijing in 2001 and 2010 respectively. MetS was defined according to the 2009 harmonizing definition.

**Results:**

A total of 2,334 participants (943 male, 1,391 female) in 2001 and 2,102 participants (848 male, 1,254 female) in 2010 completed the survey. The prevalence of MetS was 50.4% (95%CI: 48.4%–52.4%) in 2001 and 58.1% (95%CI: 56.0%–60.2%) in 2010. The absolute change of prevalence of MetS was 7.7% over the 10-year’s period (p<0.001). The syndrome was more common in female than male in both survey years. Among the five components, hypertriglyceridemia and low HDL-C had increased most, with an increase of 14.8% (from 29.4% to 44.2%) and 9.9% (from 28.3% to 38.2%) respectively. The adjusted ORs of MetS for CHD, stroke and CVD were 1.67(95%CI: 1.39–1.99), 1.50(95%CI: 1.19–1.88) and 1.70(95%CI: 1.43–2.01) respectively in 2001, and were 1.74(95%CI: 1.40–2.17), 1.25(95%CI: 0.95–1.63) and 1.52(95%CI: 1.25–1.86) respectively in 2010.

**Conclusion:**

The prevalence of MetS is high and increasing rapidly in this Chinese elderly population. Participants with Mets and its individual components are at significantly elevated ORs for CVD. Urgent public health actions are needed to control MetS and its components, especially for dislipidemia.

## Introduction

The metabolic syndrome (MetS) is a complex of interrelated risk factors for cardiovascular disease (CVD) and diabetes, resulting from obesity and insulin resistance. These factors include hyperglycemia, elevated blood pressure, hypertriglyceridemia, decreased high density-lipoprotein cholesterol (HDL-C) and obesity (particularly central adiposity). Currently available evidence suggests that MetS is associated with the development of type 2 diabetes, cardiovascular disease and an increased risk for mortality from cardiovascular disease and all causes [1]–[4].

Several studies have published information on the prevalence of MetS, however, only a few studies have reported changes of MetS, and the results were inconsistent [5]–[8]. The situation is similar in China, some studies have reported the prevalence of MetS in different groups [9]–[12], but little information exists about the change of MetS during the past decade in China, which has undergone a dramatic socioeconomic change and a succession of unhealthy lifestyles. These changes have led to an increased burden in chronic diseases [9]. Therefore, it’s urgent and important to examine changes of MetS in this rapid development and changing period in China. Our study aims to examine the prevalence and changes of MetS in an elderly population in Beijing using the 2009 harmonizing definition [13].

## Research Design and Methods

### Methods

Sampling and research methods were reported elsewhere [14]. In brief, a two-stage stratified clustering sampling method was used to conduct this population-based cross-sectional survey in participants aged ≥60 years living in Wanshoulu district, a representative urban residential area of Beijing. In 2001, a total of 2,680 participants aged 60–95 years were selected and 2,334 participants completed the survey with a response rate of 87.3%. These participants accounted for about 10% of total elderly residents in the Wanshoulu district. In 2010, we did another cross-sectional survey in the same district using the same method. A total of 2,162 participants were selected this time and 2,102 participants completed the survey. Because of the rapid socioeconomic change and huge personnel flow in China, there are only 774 participants (35%) included in both survey times.

Each participant was interviewed and completed a standardized questionnaire including a range of demographic factors, medical history, family history of CVD, and lifestyles. Specially trained nurses and observers measured the height, weight, waist and blood pressure according to the standardized protocol. Height was measured in meters (without shoes), and weight was measured in kilograms (with heavy clothing removed and 1 kg deducted for remaining garments). Body mass index (BMI) was calculated as weight in kilograms divided by the square of height in meters. Waist circumference on standing participants was measured midway between the lower rib margin and iliac crest. Two blood pressure recordings were obtained from the right arm of participants in a sitting position after 30 min of rest, and mean values were calculated. Overnight fasting blood specimens were obtained for measurement of serum lipids and glucose. Samples were sent to the central certified laboratory of Chinese PLA general hospital in less than 30 minutes.

### Definition of MetS

The 2009 harmonizing definition was used, as the presence of at least three of the following five risk factors: (1) central obesity (waist circumference ≥90 cm in Asian men and ≥80 cm in Asian women); (2) decreased HDL-C: fasting HDL-C <1.0 mmol/L in men and <1.3 mmol/L in women, drug treatment for reduced HDL-C is an alternate indicator; (3) elevated blood pressure: systolic ≥130 mmHg and/or diastolic ≥85 mmHg, antihypertensive drug treatment in a patient with a history of hypertension is an alternate indicator; (4) hypertriglyceridemia: fasting plasma triglycerides ≥1.7 mmol/L, drug treatment for elevated triglycerides is an alternate indicator; (5) hyperglycemia: fasting glucose level of ≥5.6 mmol/L (≥100 mg/dL), drug treatment of elevated glucose is an alternate indicator [13].

### Diagnosis of CVD and Diabetes

Coronary heart diseases (CHD) and stroke were defined using the WHO MONICA criteria [15]. CVD in this study was defined as having at least one of the two events. The diagnosis of CVD was based on self report and confirmed by medical records and clinical examination carried out during the survey time including electrocardiogram. Participants with fasting plasma glucose ≥7.0 mmol/l or 2-h plasma glucose≥11.1 mmol/l after oral glucose tolerance test or those who were receiving anti-diabetic medications were diagnosed with diabetes mellitus.

### Statistical Analysis

Data were entered (double entry) using Epidata 3.1. All analyses were conducted using SPSS (18.0, No. of Serial: 5076595). Reported probabilities were two-sided, with p<0.05 considered to be statistically significant.

Gender-specific prevalence of MetS was calculated using the 2009 harmonizing definition. Direct age adjustment of the data was done for participants surveyed at 2010, using the data of 2001 as reference. T test and χ2 test were used to examine differences in continuous and categorical variables, and compare the prevalence of MetS and its components by years and gender. We also did a longitudinal analysis for those who were included in both two survey times. Logistic regression was used to calculate the odds ratios (ORs) and 95% confidence intervals (CIs) for MetS.

### Ethical Considerations

The study was approved by the committee for medical ethics of the Chinese PLA General Hospital. Each participant signed an informed consent before completing the questionnaire.

## Results

A total of 2,334 participants (943male, 1,391 female) in 2001 and 2,102 participants (848 male, 1,254 female) in 2010 completed the survey. We calculated the age-adjusted value for continuous data in 2010, using 2001 as reference. General characteristics were listed in Table 1.

**Table 1:** General Characteristics in the Participants of Two Surveys at 2001 and 2010.

Characteristic	2001(n = 2334)	2010(n = 2102)	P
Mean age (yrs)	67.6(6.0)	71.2(6.6)	<0.001
Age-adjusted mean(SD)			
Waist circumference(cm)	87.6(9.4)	87.8(8.9)	0.420
BMI(kg/m^2^)	25.6(3.5)	25.0(3.4)	<0.001
Systolic blood pressure (mm Hg)	136.9(21.2)	137.1(19.2)	0.766
Diastolic blood pressure(mmHg)	77.1(10.4)	77.5 (9.7)	0.256
Fasting glucose(mmol/L)	6.1 (1.9)	6.0 (1.6)	0.103
Total cholesterol(mmol/L)	5.3(1.7)	5.3(1.0)	0.374
Triglycerides(mmol/L)	1.6(1.1)	1.7(1.0)	<0.001
HDL-C(mmol/L)	1.4(0.4)	1.4(0.4)	0.109
LDL-C(mmol/L)	3.3(1.0)	3.3(0.8)	0.846
Number (%)			
Gender			0.967
Male	943(40.4)	848(40.3)	
Female	1391(59.6)	1254(59.7)	
Education (yrs)			<0.001
0–6	967(41.4)	580(27.6)	
7–12	832(35.6)	849(40.4)	
≥13	535(22.9)	673(32.0)	
Marital status			0.141
Married	1967(84.3)	1774(84.4)	
Single or divorced	11(0.5)	20(1.0)	
Widowed	356(15.3)	308(14.7)	
Physical exercise (h/day)			<0.001
<1	615(26.3)	298(14.2)	
1–3	900(38.6)	1607(76.5)	
≥4	819(35.1)	197(9.4)	
Smoking status			<0.001
Never	1606(68.8)	1531(72.8)	
Former	375(16.1)	340(16.2)	
Current	353(15.1)	231(11.0)	
Current alcohol drinking	354(15.2)	506(24.1)	<0.001
Family histories of CVD	1180(50.6)	1308(62.2)	<0.001
CHD	784(33.6)	438(23.7)	<0.001
Stroke	378(16.2)	267(12.7)	<0.001
CVD	995(42.6)	665(31.6)	<0.001

Data are mean (SD) for continuous values or n (%) for category values;

LDL-C: low density-lipoprotein cholesterol.

### Prevalence and Changes of MetS

Table 2 showed the proportion of Chinese elderly people with MetS and individual components. The prevalence of MetS was 50.4% (95%CI: 48.4%–52.4%) in 2001 and 58.1% (95%CI: 56.0%–60.2%) in 2010 (Table 2). In both surveys, we found a higher prevalence of MetS in female than in male (P<0.001).

**Table 2:** Age-adjusted and Gender-specific Prevalence of MetS in the Participants of Two Surveys at 2001 and 2010.

	Male			Female			Total		
	2001	2010	P	2001	2010	P	2001	2010	P
**MetS components**									
Elevated blood pressure	73.6(70.8–76.4)	75.9(72.9–78.9)	0.275	73.3(71.0–75.7)	78.3(76.0–80.5)	0.003	73.4(71.6–75.2)	77.4(75.6–79.2)	0.003
Central obesity	52.1(48.9–55.3)	57.9(54.5–61.4)	0.015	77.5(75.3–79.7)	77.1(74.8–79.4)	0.799	67.2(65.3–69.1)	69.8(67.9–71.8)	0.063
Hyperglycemia	52.3(49.1–55.5)	53.0(49.5–54.5)	0.760	52.5(49.9–55.1)	49.0(46.3–51.7)	0.074	52.4(50.4–54.4)	52.1(50.0–54.2)	0.212
Hypertriglyceridemia	22.6(19.9–25.3)	38.9(35.5–42.3)	<0.001	34.1(31.6–36.6)	47.4(44.7–50.1)	<0.001	29.4(27.6–31.3)	44.2(42.1–46.3)	<0.001
Low HDL-C	16.4(14.1–18.8)	27.6(24.5–30.7)	<0.001	36.3(33.8–38.9)	44.6(41.9–47.3)	<0.001	28.3(26.5–30.1)	38.2(36.1–40.2)	<0.001
**Number of components of MetS**									
One or more	90.2(88.4–92.1)	92.8(91.0–94.6)	0.054	95.2(94.1–96.3)	94.7(93.5–95.9)	0.577	93.2(92.2–94.2)	94.0(93.0–95.0)	0.269
Two or more	68.4(65.4–71.4)	74.3(71.2–77.3)	0.007	83.1(81.1–85.1)	84.0(82.0–86.0)	0.569	77.2(75.5–78.9)	80.3(78.6–82.0)	0.011
Three or more (MetS)	40.6(37.5–43.7)	50.6(47.1–54.0)	<0.001	57.0(54.4–59.6)	62.6(60.1–65.3)	0.003	50.4(48.4–52.4)	58.1(56.0–60.2)	<0.001
Four or more	15.0(12.7–17.2)	25.8(22.8–28.9)	<0.001	29.4(27.0–31.8)	39.3(36.6–41.9)	<0.001	23.6(21.8–25.3)	34.2(32.2–36.2)	<0.001
Five	2.8(1.7–3.8)	9.8(7.7–11.9)	<0.001	9.1(7.5–10.6)	15.8(13.8–17.8)	<0.001	6.5(5.5–7.5)	13.5(12.1–15.0)	<0.001

In the 10-year’s period, the prevalence of MetS increased significantly (p<0.001), from 50.4% to 58.1%. Male had a greater increase than female, with an absolute increase of 10.0% and 5.6% respectively.

### Prevalence and Changes of Individual Components of MetS

Table 2 also demonstrated the prevalence of each component of MetS. The most common component for both male and female in both surveys was elevated blood pressure, which involved about three fourths of all the participants. Hypertriglyceridemia and low HDL-C were relatively low. There were different ranks in the five components for male and female. For male, elevated blood pressure was the highest, with central obesity and hyperglycemia followed next. While for female, central obesity and elevated blood pressure ranked first, with hyperglycemia coming third.

From 2001 to 2010, there was a dramatic increase in the prevalence of hypertriglyceridemia and low HDL-C, especially in male. Hypertriglyceridemia had an absolute increase of prevalence of 14.8%, followed by HDL-C with an absolute change of 9.9%.The increase of prevalence of elevated blood pressure was 4.0%, with 2.3% in male and 5.0% in female. The component of central obesity showed a significant increase in male (5.8%, p = 0.015) while remained unchangeable in female (−0.4%, p = 0.799). Hyperglycemia was the only component that had decreased (−0.3%, p = 0.212) during the period.

The number of components of MetS changed remarkably from 2001 to 2010. For example, individuals who had at least four of the five components of MetS increased from 23.6% to 34.2% (p<0.001) (Table 2).

### Longitudinal Analysis of MetS for the 774 Participants Included in Both Two Survey Times

Table 3 showed the prevalence of MetS for those participants include in both two survey times. The results are similar to those showed in Table 2. The prevalence of MetS has increased from 49.2% (95%CI: 45.7%–52.8%) to 58.8% (95%CI: 55.2%–61.7%) in the 10-year’s period. Female have a higher prevalence in both two survey times, but male have a greater increase (an absolute change of 12.7% for male and 8.4% for female). Of the five components, hypertriglyceridemia and low HDL-C have increased most, which are 8.9% and 8.1% respectively.

**Table 3:** Gender-specific Prevalence of MetS for Participants Included in Both Two Surveys.

	Male (n = 300)			Female (n = 474)			Total (n = 774)		
	2001	2010	P△	2001	2010	P△	2001	2010	P△
**MetS components**									
Elevated blood pressure	71.0(65.8–76.2)	76.3(71.8–80.8)	0.006	72.2(68.1–76.2)	79.9(76.5–83.3)	0.002	71.7(68.5–74.9)	79.6(76.9–82.3)	0.002
Central obesity	50.7(45.0–56.4)	60.3(54.8–65.9)	0.001	77.6(73.9–81.4)	80.8(77.2–84.4)	0.137	67.2(63.9–70.5)	72.9(69.7–76.0)	0.001
Hyperglycemia	49.7(44.0–55.4)	57.7(52.1–63.3)	0.009	48.5(44.0–53.0)	51.5(47.0–56.0)	0.239	49.0(45.4–52.5)	53.9(50.4–57.3)	0.009
Hypertriglyceridemia	24.7(19.8–29.6)	35.3(29.9–40.8)	0.001	35.4(31.1–39.8)	49.8(45.3–54.3)	<0.001	31.3(28.0–34.5)	44.2(40.7–47.7)	<0.001
Low HDL-C	14.3(10.4–18.3)	26.0(21.0–31.0)	<0.001	37.8(33.4–42.1)	43.7(39.2–48.1)	0.021	28.7(25.5–31.9)	36.8(33.4–40.2)	<0.001
**Number of components of MetS**									
One or more	90.7(87.4–94.0)	94.7(92.1–97.2)	0.050	94.5(92.5–96.6)	96.6(95.0–98.3)	0.076	93.0(91.2–94.8)	95.9(94.5–97.3)	0.005
Two or more	67.7(62.7–73.0)	78.0(73.3–82.7)	<0.001	82.3(78.8–85.7)	88.8(86.0–91.7)	0.001	76.6(73.6–79.6)	84.6(82.1–87.2)	<0.001
Three or more (MetS)	38.0(32.4–43.5)	50.7(45.0–56.3)	<0.001	56.3(51.9–60.8)	64.7(60.4–68.9)	<0.001	49.2(45.7–52.8)	58.8(55.2–61.7)	<0.001
Four or more	12.3(8.6–16.1)	25.7(20.7–30.6)	<0.001	28.7(24.6–32.8)	41.4(36.9–45.8)	<0.001	22.4(19.4–25.3)	35.3(31.9–38.6)	<0.001
Five	1.7(0.2–3.1)	10.7(7.2–14.2)	<0.001	9.7(7.0–12.4)	15.2(12.0–18.4)	0.003	6.6(4.8–8.3)	13.4(11.0–15.8)	<0.001

△ Paired χ2 test is used (McNemar method) to calculate P value.

### ORs of MetS and CVD

Table 4 showed the ORs for MetS and its individual components associated with CHD and stroke. The ORs of MetS for CHD, stroke and CVD were 1.67(95%CI: 1.39–1.99), 1.50(95%CI: 1.19–1.88) and 1.70(95%CI: 1.43–2.01) in 2001, and were 1.74(95%CI: 1.40–2.17), 1.25(95%CI: 0.95–1.63) and 1.52(95%CI: 1.25–1.86) respectively in 2010. Of the five components, the ORs of elevated blood pressure and central obesity for CVD were higher than those of the other three in 2001, which were 1.88(95%CI: 1.54–2.29) and 1.48(95%CI: 1.23–1.77). In 2010, the ORs of low HDL-C increased to1.83 (95%CI: 1.51–2.22), which were almost the same as elevated blood pressure and higher than the other three components. The ORs of hypertriglyceridemia for CVD also increased from 1.19(95%CI: 0.99–1.43) to 1.42(95%CI: 1.17–1.71) at 2001 and 2010.

**Table 4:** OR and 95% CI of CVD for MetS and its individual components.

	2001 (n = 2334)			2010 (n = 2102)		
	CHD (n = 784)	Stroke(n = 378)	CVD (n = 995)	CHD (n = 498)	Stroke (n = 267)	CVD (n = 665)
**Model 1**						
MetS	1.74(1.45–2.09)	1.51(1.20–1.90)	1.73(1.46–2.05)	1.81(1.45–2.25)	1.30(0.99–1.70)	1.57(1.29–1.90)
Individual components of MetS						
Elevated blood pressure	1.81(1.46–2.24)	1.81(1.35–2.42)	1.93(1.59–2.36)	1.92(1.42–2.59)	1.88(1.26–2.80)	1.89(1.45–2.47)
Central obesity	1.62(1.32–1.98)	1.23(0.95–1.58)	1.51(1.26–1.80)	1.52(1.20–1.93)	1.24(0.92–1.66)	1.37(1.11–1.70)
Hyperglycemia	1.30 (1.09–1.54)	1.30(1.04–1.62)	1.32(1.12–1.56)	1.33(1.08–1.63)	1.57(1.20–2.05)	1.34(1.11–1.62)
Hypertriglyceridemia	1.31(1.08–1.59)	1.09(0.86–1.40)	1.19(1.00–1.43)	1.55(1.26–1.91)	1.17(0.90–1.52)	1.45(1.20–1.75)
Low HDL-C	1.13(0.93–1.38)	1.69(1.32–2.15)	1.36(1.13–1.63)	2.05(1.66–2.52)	1.36(1.05–1.77)	1.86(1.53–2.25)
**Model 2**						
MetS	1.67(1.39–1.99)	1.50(1.19–1.88)	1.70(1.43–2.01)	1.74(1.40–2.17)	1.25(0.95–1.63)	1.52(1.25–1.86)
Individual components of MetS						
Elevated blood pressure	1.70(1.37–2.11)	1.82(1.36–2.44)	1.88(1.54–2.29)	1.83(1.35–2.48)	1.79(1.20–2.67)	1.84(1.10–2.41)
Central obesity	1.52(1.25–1.86)	1.21(0.94–1.57)	1.48(1.23–1.77)	1.49(1.18–1.89)	1.22(0.91–1.63)	1.37(1.11–1.70)
Hyperglycemia	1.29 (1.09–1.54)	1.30(1.04–1.63)	1.32(1.12–1.57)	1.29(1.05–1.60)	1.53(1.17–1.99)	1.30(1.07–1.57)
Hypertriglyceridemia	1.30(1.08–1.58)	1.08(0.84–1.38)	1.19(0.99–1.43)	1.51(1.22–1.85)	1.13(0.87–1.47)	1.42(1.17–1.71)
Low HDL-C	1.11(0.91–1.35)	1.66(1.30–2.12)	1.33(1.10–1.60)	2.00(1.62–2.47)	1.32(1.01–1.72)	1.83(1.51–2.22)

Model 1: adjusting for gender, age, education, marital status;

Model 2: adjusting for alcohol drinking, smoking, physical exercise, family histories of CVD and the 4 variables in model 1.

We also ascertained the association of MetS and CVD in the sensitivity analysis and the results were similar (Table S1). When diabetic patients (n = 362, 15.5% in 2001 and n = 652, 31.0% in2010) were excluded, the ORs for MetS associated with CVD changed but still remained significant. The adjusted ORs for MetS associated with CVD were 1.54(95%CI: 1.27–1.85) in 2001 and 1.29(95%CI: 1.02–1.63) in 2010.

## Discussion

To the best of our knowledge, this is the first study to assess the changes of prevalence of MetS in the past decade of China. It indicated that, in this Chinese elderly population, the prevalence of MetS increased significantly from 2001 to 2010, based on the 2009 harmonizing definition (absolute change 7.7%, p<0.001).

Only a few studies examined the trends of the prevalence of MetS, and the results were inconsistent, some were decreased or remained unchanged, some studies showed an increase trend but with different annual increase rate. In Korea, the prevalence of MetS increased 6.4% from 1998 to 2007 for people aged ≥20 years [6]; in Finland, the prevalence increased significantly among female (6.9%) but remained unchanged among male [7]; in US, the age-adjusted prevalence of MetS increased from 29.2% to 34.2% from National Health and Nutrition Examination Survey (NHANES)III to NHANES in 1999–2006 [8]; in Mexico, the prevalence for male stayed unchangeable between 1990–1992 and 1997–1999, but in female, the prevalence decreased from 65.4% to 59.9% [5]. Here in our study, we found an enormous increase in the prevalence of MetS among Chinese elderly. China has experienced fast economic development. As China increases its rate of modernization and becomes more urbanized, more people are likely to have sedentary lifestyles and consumption of energy-dense diets [9]. These changes are associated with lifestyle-related abnormal biochemical indexes, such as large waist circumference, dislipidemia, elevated blood pressure and hyperglycemia, and eventually causing increase in the prevalence of MetS, and this will bring an increase in diabetes and CVD and related social-economic disease burden [16], [17].

Most of the five components of MetS have increased in the 10-years’ period, while hyperglycemia and low HDL-C increased most, with an absolute change of 14.8% and 9.9% respectively. The results from the longitudinal studies also confirmed the big increase in these two components. And this finding is also consistent with other studies in China. Previous studies and efforts had paid lots of attention on hypertension and diabetes while less on dislipidemia. However, with the changes of the enormous social-demographic status, lifestyles had big transition, and Chinese people now consume more meat and fat than before. Data from the Chinese National Nutrition Survey have showed the proportion of dietary energy derived from fat increased dramatically from 19% to 28%, and the adults’ consumption of animal meat increased enormously while cereals and starchy roots declined significantly between 1991 and 2004 [16], [17]. These unhealthy lifestyles contributed to significant increase in serum cholesterol and also explained the recent rapid increase in chronic diseases in China to some extend [18]. Although the prevalence of dislipidemia increases rapidly, it didn’t get enough emphasis. A study showed the proportion of male and female in China who were aware, treated, and took control of dislipidemia was only 8.8% and 7.5%, 3.5% and 3.4%, 1.9% and 1.5%, respectively, much lower than those of hypertension and diabetes [19]. Dislipidemia is one of the most important modifiable risk factors for CVD. In order to reduce the large and increasing burden of CVD in China, we should develop more effective public health strategies for the prevention and treatment of dislipidemia, and it’s an urgent priority to reduce serum cholesterol level and the related social and medical burden in China [20].

In our study, the prevalence of MetS was much higher in female than in male, which was consistent with other research [9]. However, male increased at a faster rate, which resulted in the fact that the gender difference is reducing over the past decade. This is primarily attributable to a higher increase of central obesity and low HDL-C in male than female.

In our study, participants with MetS had significantly higher ORs of CHD, stroke and CVD. Of the five components, elevated blood pressure and central obesity not only had higher prevalence, but also had higher ORs for CVD. Hypertriglyceridemia and low HDL-C increased most in the 10-year’s period and also had an increased OR for CVD. This finding indicates that it’s important and meaningful to control both overall MetS and its individual components, so as to prevent related diabetes and CVD events.

This is a population-based cross-sectional study with strict training process and quality assurance programs. Wanshoulu District is a representative metropolitan area of the geographic and economic characteristics in Beijing, such as income levels, residential status, and lifestyle factors. The response rate was high and there was no statistically significant difference between participants who completed and those who didn’t completed the data. Consequently, the prevalence and changes of MetS can be generalized with confidence to similar population in urban Beijing, China.

Admittedly, this study had several limitations. First, it’s a two-rounded cross-sectional study, with about 35% (774 participants) of the population included in both survey times. Compared with a cohort with fix crowed, it could only calculated the prevalence of MetS in two survey years while it can’t predict the incidence accurately. But it reflected the real phenomenon in China, where big social change and huge personnel flow were happening every day [21]. To be more complete, we also did a longitudinal study for the 774 participants included in both survey times. The similar results also confirmed the increase change of MetS in China. Secondly, the study only included participants who were aged≥60 years and may have different characteristic compared with total population. But on the other hand, this aged population was more susceptible to have MetS and easily to observe the trend happened in China, so as to give us an early alert to MetS and take early prevention actions.

In summary, the present study showed a high and increasing prevalence of MetS in Chinese elderly population living in urban Beijing between 2001 and 2010, especially for dislipidemia components. These findings indicate that MetS is becoming major public health problems in China. Clearly, without prevention and aggressive treatment of these conditions, the potential socioeconomic and medical burden in China could be overwhelming.

## Supporting Information

Table S1 **Table S1 showed OR and 95% CI of CVD for MetS and its individual components at 2001 and 2010 excluding subjects with diabetes.**
(DOC)
