# Supplementary material for: Increasing Prevalence of Metabolic Syndrome in a Chinese Elderly Population: 2001–2010
Source: PLoS One. 2013 Jun 18;8(6):e66233. doi: 10.1371/journal.pone.0066233 (PMC3688874; doi:10.1371/journal.pone.0066233)
Supplement: Table S1 — showed OR and 95% CI of CVD for MetS and its individual components at 2001 and 2010 excluding subjects with diabetes. (DOC) [file pone.0066233.s001.doc]

**Table S1.** **OR and 95% CI of CVD for MetS and its individual components excluding subjects with diabetes**

|  | 2001 | | |  | 2010 | | |
| --- | --- | --- | --- | --- | --- | --- | --- |
|  | CHD (n=632) | Stroke(n=291) | CVD (n=797) |  | CHD (n=300) | Stroke (n=160) | CVD (n=406) |
| **Model 1** |  |  |  |  |  |  |  |
| MetS | 1.57(1.59-1.91) | 1.40(1.09-1.81) | 1.56(1.29-1.88) |  | 1.66(1.28-2.16) | 1.30(0.99-1.70) | 1.38(1.09-1.74) |
| Individual components of MetS |  |  |  |  |  |  |  |
| Elevated blood pressure | 1.69(1.34-2.12) | 1.77(1.28-2.44) | 1.82(1.47-2.26) |  | 1.74(1.22-2.47) | 1.64(1.03-2.61) | 1.71(1.25-2.33) |
| Central obesity | 1.53(1.23-1.91) | 1.12(0.85-1.47) | 1.40(1.14-1.72) |  | 1.59(1.19-2.13) | 1.34(0.93-1.94) | 1.41(1.09-1.82) |
| Hyperglycemia | 1.17 (0.97-1.42) | 1.07(0.83-1.38) | 1.14(0.95-1.37) |  | 0.94(0.72-1.24) | 1.34(0.99-1.95) | 0.96(0.75-1.23) |
| Hypertriglyceridemia | 1.22(0.99-1.51) | 1.01(0.76-1.34) | 1.09(0.89-1.34) |  | 1.83(1.41-2.38) | 1.11(0.79-1.56) | 1.57(1.24-1.99) |
| Low HDL-C | 1.08(0.87-1.35) | 1.69(1.29-2.22) | 1.30(1.05-1.61) |  | 2.28(1.75-2.98) | 1.30(0.92-1.84) | 1.94(1.52-2.47) |
| **Model 2** |  |  |  |  |  |  |  |
| MetS | 1.51(1.24-1.84) | 1.38(1.07-1.78) | 1.54(1.27-1.85) |  | 1.56(1.20-2.03) | 1.02(0.73-1.42) | 1.29(1.02-1.63) |
| Individual components of MetS |  |  |  |  |  |  |  |
| Elevated blood pressure | 1.58(1.26-1.99) | 1.72(1.24-2.38) | 1.77(1.42-2.19) |  | 1.66(1.17-2.36) | 1.56(0.98-2.49) | 1.62(1.18-2.21) |
| Central obesity | 1.45(1.17-1.80) | 1.10(0.83-1.45) | 1.39(1.13-1.70) |  | 1.54(1.15-2.07) | 1.31(0.90-1.89) | 1.36(1.05-1.76) |
| Hyperglycemia | 1.17 (0.96-1.42) | 1.07(0.83-1.38) | 1.14(0.95-1.37) |  | 0.92(0.70-1.22) | 1.36(0.96-1.91) | 0.94(0.73-1.20) |
| Hypertriglyceridemia | 1.20(0.97-1.49) | 1.01(0.76-1.33) | 1.09(0.89-1.34) |  | 1.74(1.34-2.26) | 1.06(0.76-1.49) | 1.48(1.16-1.88) |
| Low HDL-C | 1.07(0.85-1.33) | 1.67(1.27-2.19) | 1.28(1.04-1.58) |  | 2.20(1.68-2.88) | 1.26(0.89-1.79) | 1.87(1.46-2.39) |

*Model 1: adjusting for gender, age, education, marital status;*

*Model 2: adjusting for alcohol drinking, smoking, physical exercise, family histories of CVD and the 4 variables in model 1.*
